# Correction: A Method for Modeling Growth of Organs and Transplants Based on the General Growth Law: Application to the Liver in Dogs and Humans

**DOI:** 10.1371/journal.pone.0105483

**Published:** 2014-08-06

**Authors:** The PLOS ONE Staff

Figure 4 is incorrect. The authors have provided a corrected version here.

**Figure 4:**
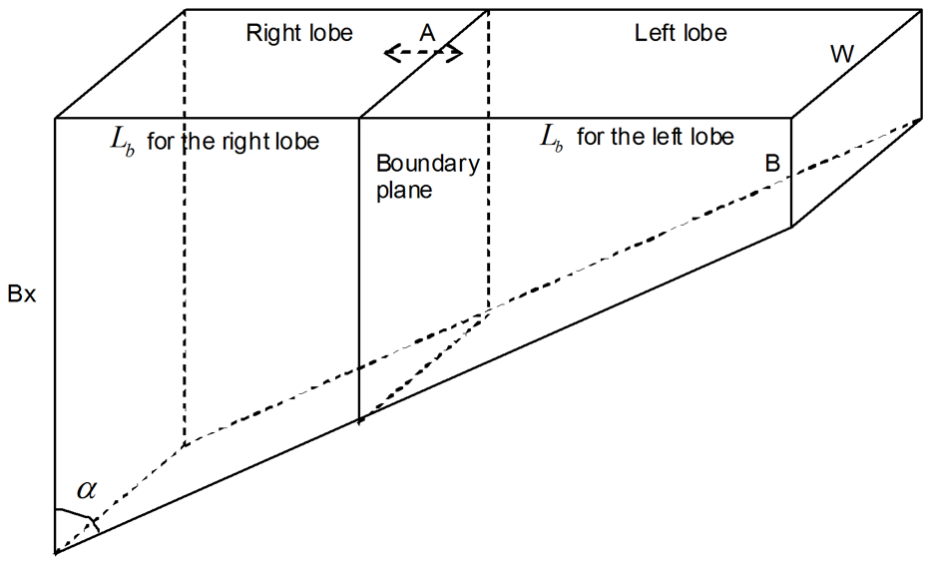
Geometric model of a human liver. The boundary plane defines the initial volume of the transplanted lobe. It can be shifted along the direction of arrow A.
